# Barium Oxide Doped Magnesium Silicate Nanopowders for Bone Fracture Healing: Preparation, Characterization, Antibacterial and In Vivo Animal Studies

**DOI:** 10.3390/pharmaceutics14081582

**Published:** 2022-07-29

**Authors:** Mostafa Mabrouk, Ghadha Ibrahim Fouad, Hanan H. Beherei, Diganta Bhusan Das

**Affiliations:** 1 Refractories, Ceramics and Building Materials Department, Advanced Materials, Technology and Mineral Resources Research Institute, National Research Centre (NRC), 33 El Bohouth St. (Former EL Tahrir St.), Dokki, Giza 12622, Egypt; hananh.beherei@gmail.com; 2 Department of Chemical Engineering, Loughborough University, Loughborough LE11 3TU, Leicestershire, UK; 3 Department of Therapeutic Chemistry, Pharmaceutical and Drug Industries Research Institute, National Research Centre, 33 El-Bohouth St., Dokki, Cairo 12622, Egypt; ghadhaibrahim@gmail.com or

**Keywords:** barium doping, magnesium silicate nanopowders, osteogenesis, bone fractures healing

## Abstract

Magnesium silicate (MgS) nanopowders doped with barium oxide (BaO) were prepared by sol-gel technique, which were then implanted into a fracture of a tibia bone in rats for studying enhanced in vivo bone regeneration. The produced nanopowders were characterized using X-ray diffraction (XRD), Fourier transform infrared spectra (FTIR), scanning electron microscope with energy-dispersive X-ray spectrometry (SEM-EDX) and transmission electron microscope (TEM). Mechanical and bactericidal properties of the nanopowders were also determined. Increased crystallinity, particle diameter and surface area were found to decrease after the BaO doping without any notable alterations on their chemical integrities. Moreover, elevated mechanical and antibacterial characteristics were recognized for higher BaO doping concentrations. Our animal studies demonstrated that impressive new bone tissues were formed in the fractures while the prepared samples degraded, indicating that the osteogenesis and degradability of the BaO containing MgS samples were better than the control MgS. The results of the animal study indicated that the simultaneous bone formation on magnesium biomaterial silicate and barium MgS with completed bone healing after five weeks of implantations. The findings also demonstrated that the prepared samples with good biocompatibility and degradability could enhance vascularization and osteogenesis, and they have therapeutic potential to heal bone fractures.

## 1. Introduction

Bone fractures occur as a result of a load that exceeds the bone strength or the cyclic activity of the loads [1]. Therefore, bone fracture healing presents a significant challenge in orthopedic surgery. It is expected that the total number of hip fracture cases will increase to 6.26 million by 2050 [2]. In addition, there are non-unions or delayed bone fracture cases depending on certain geometric, mechanical and biological factors, which require several different types of fixations to stabilize the fractures [3]. As stated in [4] “bone-fracture healing” is a complicated process that is aimed at regenerating mineralized tissue at the site of the bone defects, where the biological and mechanical processes are similar to that of the soft tissue healing but without the formation of scar tissue [5]. It also involves consequent events starting with hematoma formation, inflammation, cartilaginous formation, vascularization, mineralization and hard-callus formation which will be remodeled by osteoclasts activity [6]. Bone fracture repairs of bone defects and non-unions are governed by different structural and functional mechanisms, as discussed in [7].

Normally, the broken bone tissues are regenerated spontaneously, but under some conditions, therapeutic interventions are required to stimulate the healing response [8]. Several factors can enhance the bone healing capacity such as the use of biomaterials for reconstructing the injured area and therapeutic agents for accelerating the bone reconstruction [9,10]. Nanomaterials (NMs), mimicking the nano-features of the bones, are promising candidates because they can provide smart mechanical functions for effective bone fracture healing [11] and improved bone cell functions compared to their micron-sized counterparts [12,13]. Currently, applications of NMs in regenerative medicine and bone fracture repair are of great interest [14]. Thus, the “ideal” NMs used for bone-healing process should be biocompatible, non-toxic and biodegradable. In addressing these challenges, we attempt to find a novel alternative in this work for improved bone fracture repair and fixation through accelerating the bone healing process, thereby decreasing the healing period, reducing the cost of treatment and helping patients to recover quickly [15].

Silicate-based NMs represent a novel type of biomaterials that exhibit a highly specific surface area, which can accelerate the kinetics of apatite deposition, thus promoting the bone-healing bioactivity [16]. In addition, their degradable products demonstrate weak alkalinity, which is favorable for cellular growth and tissue regeneration [17,18]. Furthermore, they are capable of enhancing vascularization of osteoblastic cells through stimulating the upregulation of vascular endothelial growth factor (VEGF) from osteoblastic cells and induce angiogenesis that could stimulate bone formation [19]. Additionally, silicate biomaterials are able to enhance osteogenic differentiation of osteoblastic cells, which results in new bone formation [20].

Trace cations (i.e., Mg^2+^, Ba^2+^, Sr^2+^, Fe^2+^/^3+^, Zn^2+^, Cu^2+^, etc.) and anions (i.e., CO_3_^2^^−^, F^−^, SiO_4_^4^^−^, etc.) play vital roles in bone regeneration [21]. Barium (Ba^2+^) is one of the substitution cations that could be used, though non-essential but beneficial, in bone repair and regeneration (osteogenesis); Ba^2+^ ions stimulated the in vitro apatite formation of calcium phosphate on the bioceramics surfaces synthesized through the wet precipitation method [22]. However, the doping effect of BaO on the magnesium silicate (MgS) family and it’s in vivo studies are not explored in detail. In addressing this, the current study is aimed at comparing the bone healing potential as well as the nanotoxicity of MgS nanopowders, free from and doped with different concentrations of BaO. These will include evaluations of their physicochemical, morphological, mechanical and antibacterial properties alongside the in vivo studies, as discussed in detail in the following sections.

## 2. Materials and Methods

MgS nanopowders were prepared by a sol-gel technique. In particular, tetraethyl orthosilicate (TEOS: 98%, molecular weight 208.33 g/mol, ACROS Organics, Germany) was hydrolyzed in co-solvent of ethanol and distilled water (1:1 *v/v* ratios) and allowed to stir for 30 min. Upon complete hydrolysis of TEOS, the magnesium nitrate hexahydrate (H_12_MgN_2_O_12_, molecular weight 256.41 g/mol, Santa Cruz, CA, USA) was dissolved. The mixture was stirred for 60 min and then dried at 60 °C for 18 h. The obtained nanopowder was calcined at 600 °C for 2 h. In order to perform the doping process, before the drying step, barium nitrate (Ba(NO_3_)_2,_ molecular weight 261.34g/mol, Santa Cruz Biotechnology, Santa Cruz, CA, USA) was added to the MgS solution on the expense of Mg with BaO_2_ with different *w/w* ratios (3, 5 and 7). The weight percentages were calculated according to the constituents’ oxides. The ratios of the produced nanopowders in oxide form are shown in Table 1.

### 2.1. Characterization Techniques

#### 2.1.1. XRD Examinations

The X-rays diffraction (XRD) method was utilized to determine the significance of the crystallinity of the BaO-doped MgS nanopowders. The test was carried out utilizing the BRUKER AXS D8 ADVANCE model, which isappended with Ni-filter Cu-Kα illumination at 40 Kv and 40 mA, (Karlsruhe, Germany). In order to identify the obtained crystalline phases, data were matched with JCPDS database software.

#### 2.1.2. FTIR Examinations

To figure out the influence of BaO on the chemical entirety of the prepared MgS nanopowders, they were applied to infrared spectra, utilizing a Perkin Elmer Fourier Transform Infrared spectrophotometer (FTIR; model FT/IR-6100 sort A). The spectra were recorded at wave number ranging between 400–4000 cm^−1^ with a resolution of 4 cm^−1^ and 20 scans per spectrum. The specimens were prepared for examination by blending MgS nanopowders with KBr.

#### 2.1.3. Morphological and Elemental Analyses

##### Scanning Electron Microscopy Examinations

The particle morphology and size of the resulting nanopowders were analyzed using Scanning Electron Microscopy (SEM) (SEM/EDXA model FEJ quanta 250 Fei-Holland) at 15 kVm. Each sample was fixed on the scanning stubs using carbon double face tape then coated with gold and monitored by the SEM. At the same time, the elemental analysis and their distribution throughout the nanopowder samples were investigated by energy-dispersive X-ray microanalysis (EDAX). 

##### Transmission Electron Microscopy Examinations

The dispersed morphology and size of the resulted nanopowder was analyzed by transmission electron microscope (TEM). Each nanopowder sample was dispersed in ethanol and sonicated for 15 min using an ultrasonic path. Then, the copper grid was submerged in the previous dispersion, dried in air and these grids were monitored by the TEM. In addition, the diffraction patterns of the nanopowder samples were also recorded. 

### 2.2. Surface Area Measurements

Nitrogen adsorption-desorption measurements were carried out at 77.35 K on a Nova Touch LX4 Quantachrome, USA to determine the BET surface area parameters. Before the measurements, samples were degassed under high vacuum for a specific time. Liquid nitrogen was used to cool samples and analyzed by measuring the volume of gas (N_2_) adsorbed at specific pressures. The pore volume was measured from the adsorption branch of the isotherm curve at P/P_o_ = 0.995, assuming a complete pore saturation.

### 2.3. Mechanical Properties Analyses

Mechanical features of the MgS nanopowders before and after doping with BaO were analyzed by universal testing machine (Zwick Roell-Z0.5 TH Mechanical Test Equipment, Ulm, Germany). In detail, each sample was compressed into a cylindrical shape with dimensions of 20 mm height and 10 mm diameter using a hydraulic press and a stainless-steel mold under 1 ton compression force for 30 s. The cylinders were then tested using universal testing machine at a specific driven press (10 tons/min) anda compression rate of 0.5 mm/min by10 kN load cell. The test was conducted for triple cylinders of each sample and the average values were taken in order to confirm the outcomes.

### 2.4. Antibacterial Investigations

Agar diffusion method was used to assess the antibacterial capacity of the MgS nanoparticles free from and doped with BaO. The bacteria used in this study were Gram-negative bacterial strains (*Escherichia coli* ATCC-25922 and *Pseudomonas aeruginosa* ATCC-27853) and fungi (*Candida albicans* ATCC-10231). These micro-organisms were obtained from the American Type Culture Collection (ATCC, Rockville, MD, USA) and Northern Utilization Research and Development Division, United State Department of Agriculture, Peoria, IL, USA (NRRL). The bacterial cultures were prepared according to a previously reported work [23]. This was then managed into sterile petri-dishes named with the bacterial species. Liquid sterile Muller–Hinton agar was utilized to aseptically fill the plates and gently shake the microorganisms to scatter them homogeneously into the medium in the plate. The agar plates were permitted to harden after which similar loads of 15 MgS nanopowders were placed as disks on the agar surfaces, then the plates were permitted to diffuse for 2 h at 4 °C. All plates were kept at 37 °C for 24 h for bacterial strains, after which it was maintained at 28–30 °C for 48 h for the contagious strain. Diameters of the cleared bacterial zones in the petri dishes were estimated in millimeters [24].

### 2.5. Animal Studies

#### 2.5.1. In Vivo Animal Studies

The experimental design included 20 adult male Wistar rats weighing 200–250 g. The rats were housed at the animal house of the National Research Center (NRC) in separate cages, where each cage contained two rats. Rats were allowed to acclimatize for twoweeks before experimental use and maintained at a constant temperature (22 ± 1.0 °C) and humidity (55 ± 5%) in an artificial 12h light/dark cycle. Rats were kept under good ventilation and were provided with commercial rat chow and tap water throughout the experimental period. The animal protocol was conducted according tothe National Research Council’s Guide for the Care and Use of Laboratory Animals (NIH Publication No. 8023, revised in 1978), and experimental procedures were approved by the Ethical Committee of NRC, Egypt (approval no. 18060).

#### 2.5.2. Surgical Operations and Experimental Design 

Prior to the surgical procedure, the chosen rats were administrated an intraperitoneal injection of chloral hydrate (10%, 3mL/kg) to induce anesthesia. The hind limbs were shaved, disinfected with Betadine^®^ (Povidone-iodine) and 70% alcohol over three alternating cycles in preparation for incision. Subsequently, a skin incision of about 1–1.5 cm was performed using a scalpel without disturbing the muscles underneath the skin. The soft tissues were then dissected carefully till the tibia. Once the tibia bone was exposed, a fracture in the middle of the exposed part was created in all rat groups and the three different formulations of the bone cements (mixture of 50 mg of nanopowders and 100 µL of fresh phosphate buffer saline to form a homogenous paste to avoid powder leakage) were implanted at the site of the fracture using a surgical spatula. The tibia of the Sham control group was fractured in the same area without implantation. The rats were allocated into four groups (*n* = 5) according to the formulation of bone cement, as shown below:

Group (1): Sham control group—bone fracture was left empty and untreated.
Group (2): Pure MgS group—bone fracture was stuffed with MgS (50mg weight per animal).
Group (3): MgS Ba-doped (3% wt) group—bone fracture was stuffed with MgS nanopowders doped with 3% wt percentage of BaO.
Group (4): MgS Ba-doped (5% wt) group—bone fracture was stuffed with MgS nanopowders doped with 5% BaO.

**Figure 1:**
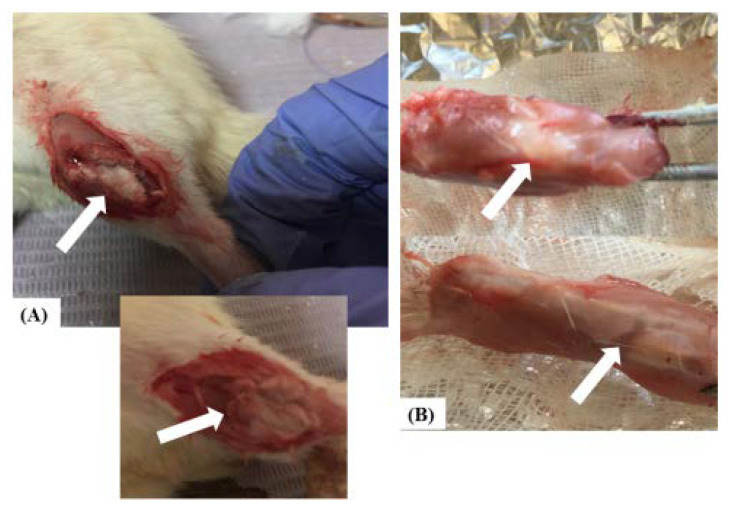
The surgical procedures that involved the application of three different formulations of MgS nanopowders at the site of fractures in the tibias of rats of different experimental groups: (**A**) Surgical procedure and implantation of different MgS nanopowders; (**B**) Macroscopic examination of post-operative healed bones. The white arrows in the images refer to either the site of fracture or healing in (**A**) and (**B**), respectively.

After the completion of procedures, the muscles and skins were repositioned and sutured using absorbable suture material PGA 3/0 (26 mm) surgical thread. Postoperatively, the rats were injected intramuscularly by dose of antibiotic (Flumox; 0.84 mg/100 g of body weight) and analgesic/anti-inflammatory agent (ceftriaxone HCl; 25 mg/kg g of body weight), once daily for 5consecutive days starting immediately after the operation, and the wound was daily sterilized with Povidoneiodine solution (10% *w/v*) and covered with a sterile cotton gauze (Figure 1).

#### 2.5.3. Clinical Observation

The rats were clinically observed on a daily basis, checked for any signs of lameness, infection or bleeding and their use of tibia was evaluated. Additionally, the injured areas were examined for edema, inflammation, swelling and pain on palpation [25].

#### 2.5.4. Blood and Tissue Preparation

After 5 weeks of applying three formulations of MgS samples, all rats were anaesthetized and euthanized to collect their tibia and liver samples. Blood samples were withdrawn, and serum was separated by centrifugation at 3000 rpm for 15 min. Then, thoracoabdominal incision was made and livers of all rats were dissected, removed and half of all extracted livers were immersed in 10% formol saline for histopathological examination. The treated tibia of different groups of rats were rapidly harvested and subsequently immersed in 10% formol saline for histopathological examination.

### 2.6. Biochemical Assays

#### 2.6.1. Determination of Serum Transaminases and Alkaline Phosphatase

Serum alanine amino transaminase (ALT) and aspartate amino transaminase (AST) were estimated colorimetrically [26]. Serum alkaline phosphatase (ALP) was measured spectrophotometrically [27]. Enzymatic activities of AST and ALT were expressed as Units (U)/mL, where ALP activities were expressed as IU/L.

#### 2.6.2. Evaluation of Serum Levels of Oxidative Stress Markers

Serum total antioxidant capacity (TAC) was measured spectrophotometrically and was expressed as mM/L [28]. Serum malondialdehyde (MDA) content was measured spectrophotometrically at 534 nm and was expressed as nmol/mL [29]. The reduced levels of serum glutathione (GSH) were estimated spectrophotometrically at 405 nm and expressed as mg/dL [30]. Serum catalase (CAT) activity was estimated spectrophotometrically at 510 nm and expressed as units/L [31].

### 2.7. Histological Examination of Tibial and Liver Samples

As discussed earlier, the experimental animals were euthanized at five weeks post-operation and implantation of different formulation of MgS. Samples were taken from the tibia and liver of rats in different groups and fixed in 10% formol saline for 24 h. Washing was done in tap water; then, serial dilutions of alcohol (methyl, ethyl and absolute ethyl) were used for dehydration. Specimens were cleared in xylene and embedded in paraffin at 56 °C in a hot air oven for 24 h. Paraffin bees wax tissue blocks were prepared for sectioning at 4 microns thickness by sledge microtome. The obtained tissue sections were collected on glass slides, deparaffinized and stained by hematoxylin and eosin for examination using the light electric microscope [32].

### 2.8. Statistical Analyses

The collected data were presented as mean ± SEM (standard error of the mean) for *n* = 5 rats of each group. The data were subjected to simple one-way analysis of variance (ANOVA) using SPSS^®^ software, version 19 (SPSS Inc., IBM Corporation, Armonk, NY, USA). Duncan’s multiple range test was used to differentiate between significant means at *p*-value < 0.05.

## 3. Results

### 3.1. XRD Analysis

**Figure 2:**
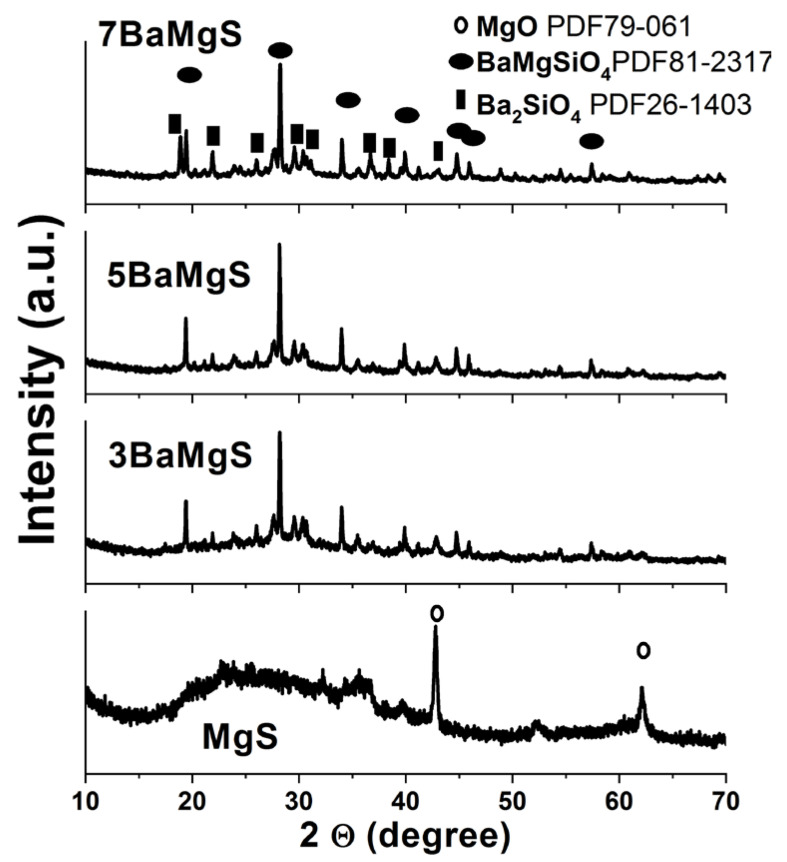
XRD curves of the prepared MgS samples before and after doping with barium.

The physical nature of the MgS nanopowders before and after doping with BaO was investigated using XRD technique. As can be seen in Figure 2, pure MgS possessed a broad amorphous hump with two small crystalline peaks. These peaks were found to correspond to those for MgO when they were compared with XRD data base. The presence of these two peaks could be owing to the fact that some of the Mg content were segregated and formed MgO. However, the two peaks disappeared upon the addition of BaO dopant to the system and also resulted in an increase in the degree of crystallinity of the produced nanopowders. The degree of crystallization was found to be dependent on the dopant concentration, where the higher the BaO concentration, the higher the obtained crystallinity. The obtained phases were found to be BaMgSiO_4_ (PDF81-2317) and Ba_2_SiO_2_ (PDF26-1403) after matching the obtained results with the XRD database. XRD results demonstrated the effective role of barium doping in MgS system to increase the crystallinity of the prepared nanopowders [22,33].

### 3.2. FTIR Characterization

**Figure 3:**
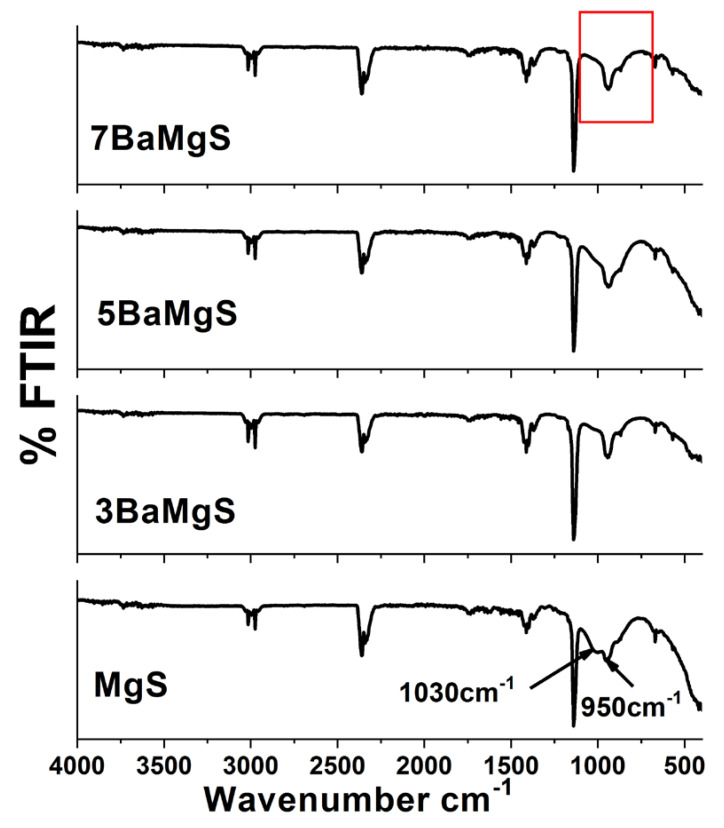
FTIR spectra of the prepared MgS samples before and after doping with Barium.

The effects of the dopant presence and concentrations on the prepared MgS were analyzed using FTIR analysis. The MgS pure nanopowders exhibited stretching vibration band at 1641 cm^−1^, which is ascribed to the zeolite water. The presence of an Si–O bending group was confirmed by observed bands at1018 cm^−1^ and 619 cm^−1^. In addition, Si–O stretching vibration entity was also noted at 1108 cm^−1^and 916 cm^−1^ [34]. Mg–O stretching vibration group was confirmed by the appearance of band at 459 cm^−1^. The effects of BaO doping can only be observed from the changes in the intensities of bands locate at 950 and 1030 cm^−1^ (Figure 3). The band at 1030 cm^−1^ disappeared gradually with an increase of BaO concentration. This band could correspond to the presence of H^+^ ion that is associated with MgO presence, which diminishes with the presence of BaO, and this result is consistent with the XRD results. In addition, the band at 930 cm^−1^ was found to be positively affected with BaO concentration; the higher BaO concentrations, the higher intensity recorded for this band. The FTIR results confirmed the presence of BaO in the nanopowders [22,33].

### 3.3. Morphological and Elemental Analyses Results

#### 3.3.1. Scanning Electron Microscopy Examinations Results

**Figure 4:**
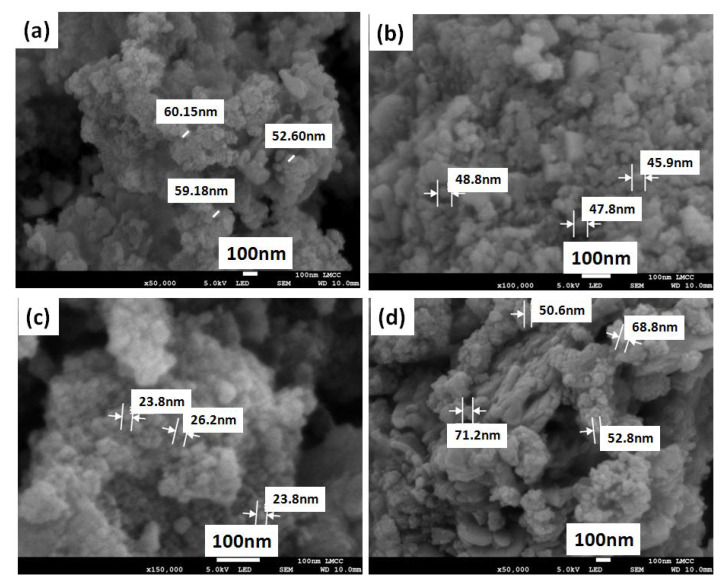
SEM images of (**a**) pure MgS, (**b**) MgS doped with 3% *w/w* BaO, (**c**) MgS doped with 5% *w/w* BaO and (**d**) MgS doped with 7% *w/w* BaO.

The effect of the barium doping on the final morphology and size of the prepared nanopowders was determined by using SEM. Generally, the morphology of the obtained nanopowders before and after doping with BaO showed typical agglomerations of glass-ceramic nanoparticles and that was consistent with the XRD results. Moreover, the pure MgS nanopowders exhibited particle size in the range of 50–60 nm (Figure 4a). Particle size was found to be decreased (45–50 nm and 20–27 nm) for the BaO-doped MgS nanopowders for both 3 and 5% *w/w*, respectively (Figure 4c,d). However, the higher BaO concentration of 7% *w/w* caused particle size increment (50–75 nm), as can be seen in Figure 4d. This could be explained by the hypothesis that as the BaO concentration increased the crystallinity degree alongside with the particles fusions, as confirmed above by the XRD results, which caused the increment of the particle size.

**Figure 5:**
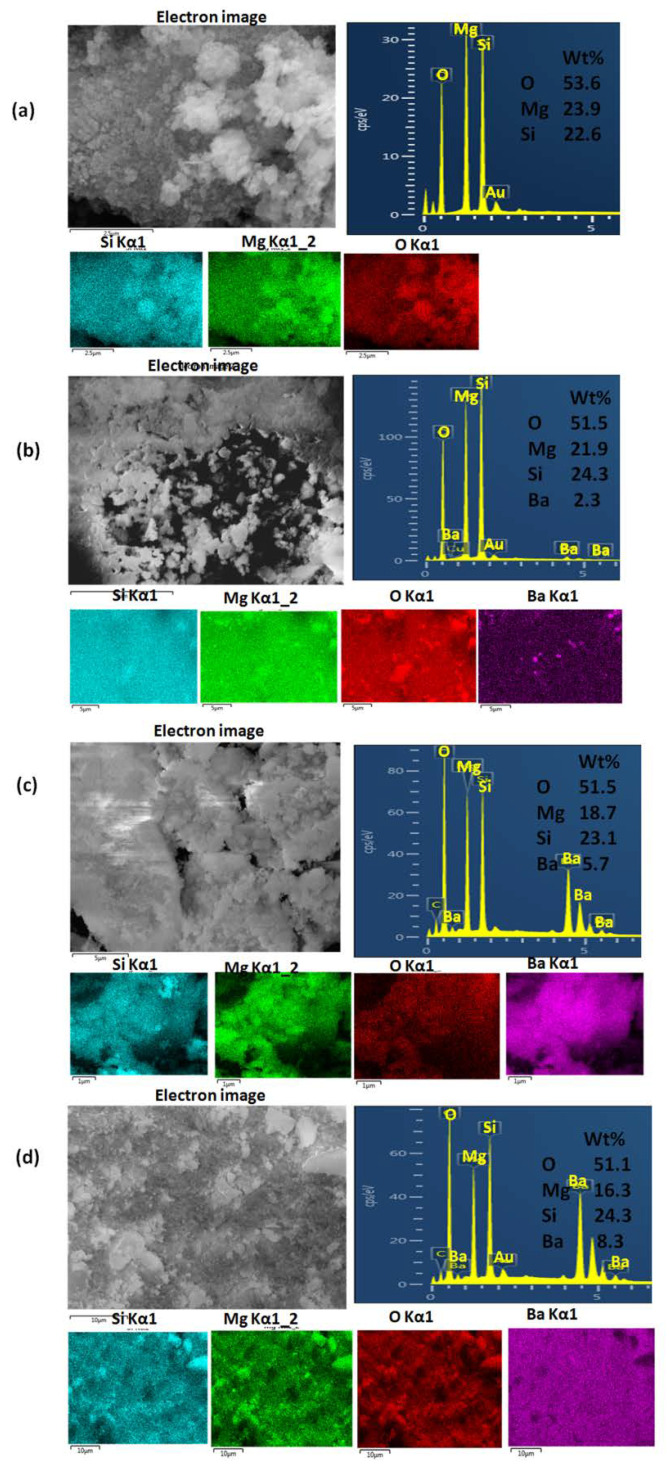
The electronic images, EDAX analysis chart and elemental mapping of (**a**) pure MgS, (**b**) MgS doped with 3% *w/w* BaO, (**c**) MgS doped with 5% *w/w* BaO and (**d**) MgS doped with 7% *w/w* BaO.

Furthermore, EDAX analysis and elemental mapping of the prepared MgS nanopowders before and after doping with BaO were investigated employing an EDAX analyzer as illustrated in Figure 5. These illustrations confirmed the presence of the Mg and Si elements in all samples as well as the BaO doping with different concentrations, which are very close to theoretical ones. Moreover, distribution homogeneities were confirmed for the dopant and the parent materials, as can be observed from the mapping images.

#### 3.3.2. Transmission Electron Microscopy Examinations Results

**Figure 6:**
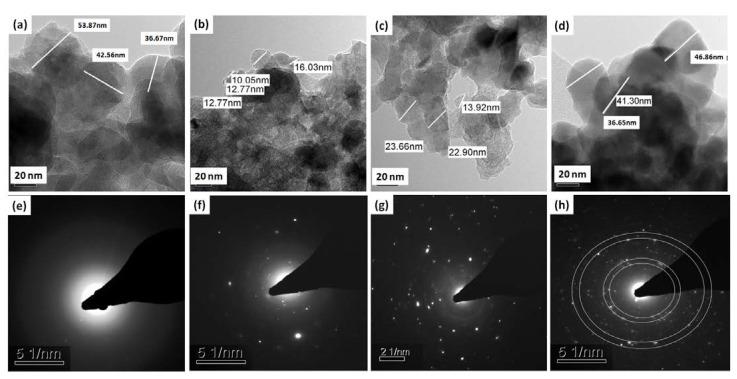
TEM images and corresponding diffraction patterns of (**a**,**e**) pure MgS, (**b**,**f**) MgS doped with 3% *w/w* BaO, (**c**,**g**) MgS doped with 5% *w/w* BaO and (**d**,**h**) MgS doped with 7% *w/w* BaO.

The morphology and particle size of the dispersed nanopowders were determined by TEM images, as shown in Figure 6. In addition, the corresponding diffraction patterns of the MgS samples were also investigated. It can be noted that the particle size and crystallinity degree of the prepared nanopowders are highly affected by presence of BaO dopant and its concentration. Furthermore, the diffraction patterns of all samples confirmed the gradual increase in the crystallinity degree that takes place with increased concentration of BaO and become more ordered and pronounced with the highest concentration (BaO7% *w/w*). 

### 3.4. Surface Area Measurements Results

Surface area parameters were evaluated for all the prepared samples using BET surface area measurements, as listed in Table 2. Major negative effects for BaO presence and concentration were observed when surface area was decreased from 122.63 ± 0.54 m²/g (pure MgS) to 37.95 ± 0.30 m²/g (7% *w/w* BaO). This might be consistence with the results of SEM and TEM images; however, samples doped with3 and 5% *w/w* BaO showed smaller particle size, which are supposed to possess a higher surface area value. This could be explained by that the presence of BaO has increased the cementing property of these materials, which had increased their agglomeration and decreased their surface area.

### 3.5. Mechanical Properties

**Figure 7:**
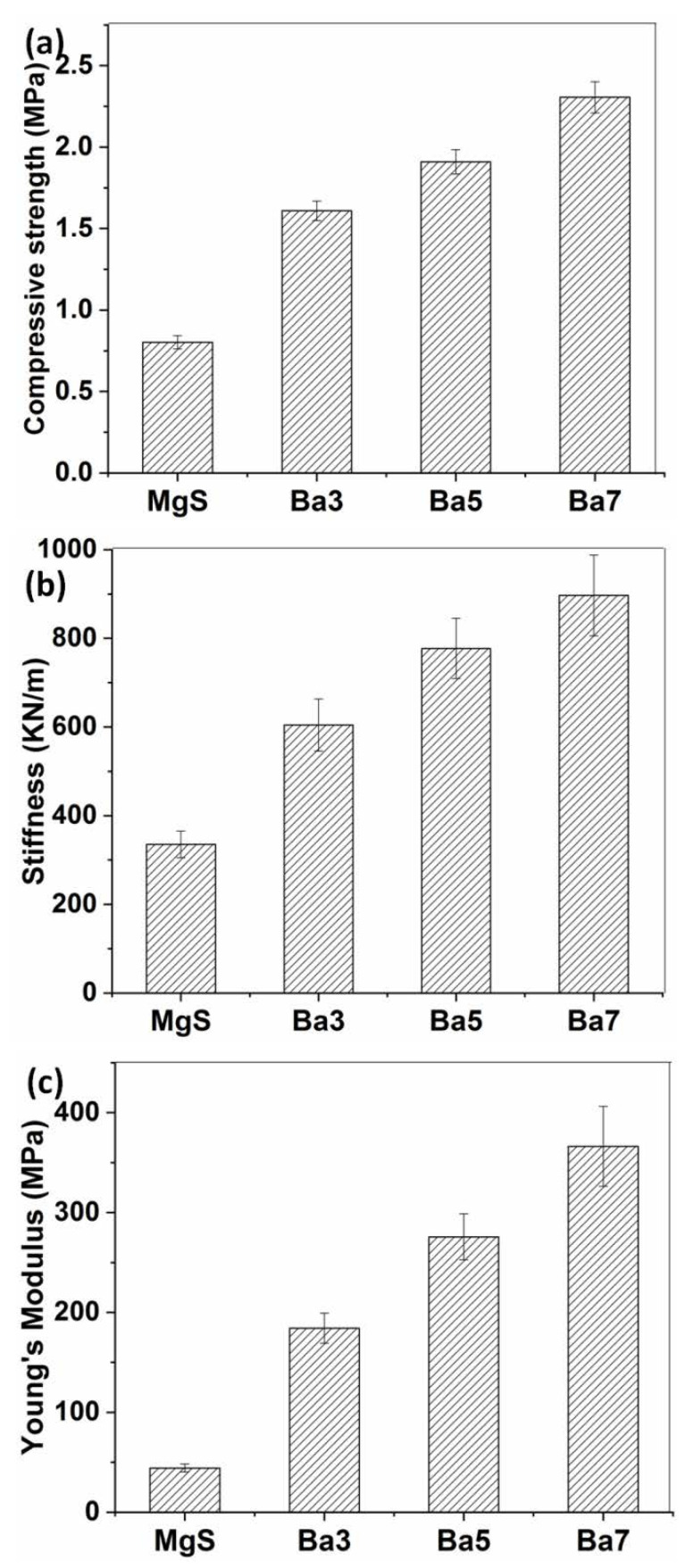
Mechanical properties of MgS before and after doping with BaO. (**a**) Compressive strength, (**b**) stiffness and (**c**) Young’s modulus.

The mechanical properties of the achieved MgS nanopowders before and after doping with BaO were assessed using universal testing machine and the results were represented in Figure 7. It was obvious that BaO doping has a positive influence on all the mechanical parameter recorded by this test. In detail, compressive strength of the pure MgS was raised from 0.8 MPa to 1.6 MPa at a low concentration of BaO doping (Figure 7a). Furthermore, this increment was maintained and enhanced with the increase in the BaO concentrations up to 7% to record a compressive strength (2.3 MPa). The stiffness and Young’s modulus exhibited the same trend as the compressive strength was relatively improved as the BaO concentration increased their values.

### 3.6. Antibacterial Investigations Results

**Figure 8:**
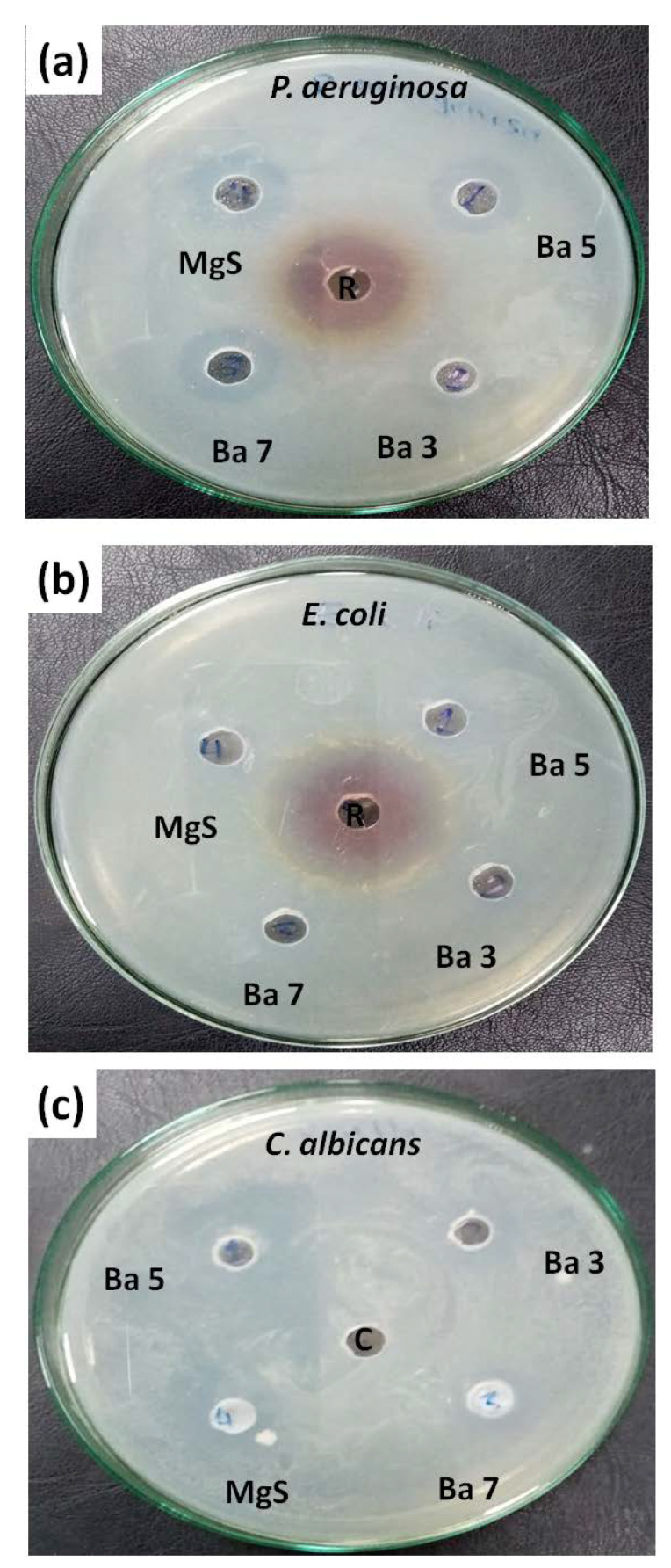
Antibacterial capabilities of MgS before and after doping with BaO against bacterial strains (**a**) *Pseudomonas aeruginosa*, (**b**) *Esherichia coli* and (**c**) *Candida albicans*.

The obtained samples were investigated against the mentioned strains in the methodology section to determine their antibacterial capabilities as illustrated in Figure 8. All the samples demonstrated antibacterial capabilities against the tested bacterial strains, even the pure MgS nanopowders. Practically, pure MgS nanopowders demonstrated disinfected zones with diameters of 18, 12 and 14 mm in the strains *Pseudomonas aeruginosa*, *Esherichia coli* and *Candida albicans*, respectively. The samples doped with BaO exhibited very similar results regardless the BaO concentrations. The recorded diameters of the disinfected zones by BaO-doped MgS samples were in the range of 18–16 mm, which was comparable to the diameters of the disinfected zones observed for Rifampicin (A) and Cyclohexamide (C), which are very potent antibacterial and antifungal agents.

### 3.7. In Vivo Animal Study

#### 3.7.1. Clinical Evaluation 

All rats in different experimental groups regained consciousness post-operation with slight swelling observed at the fracture site. There was no mortality throughout the experimental period. Ten days after surgery, the rats could move their hind limbs with no signs of local inflammation or pain.

#### 3.7.2. Hepatotoxicity Indices in Tibia-Fractured Rats 

**Figure 9:**
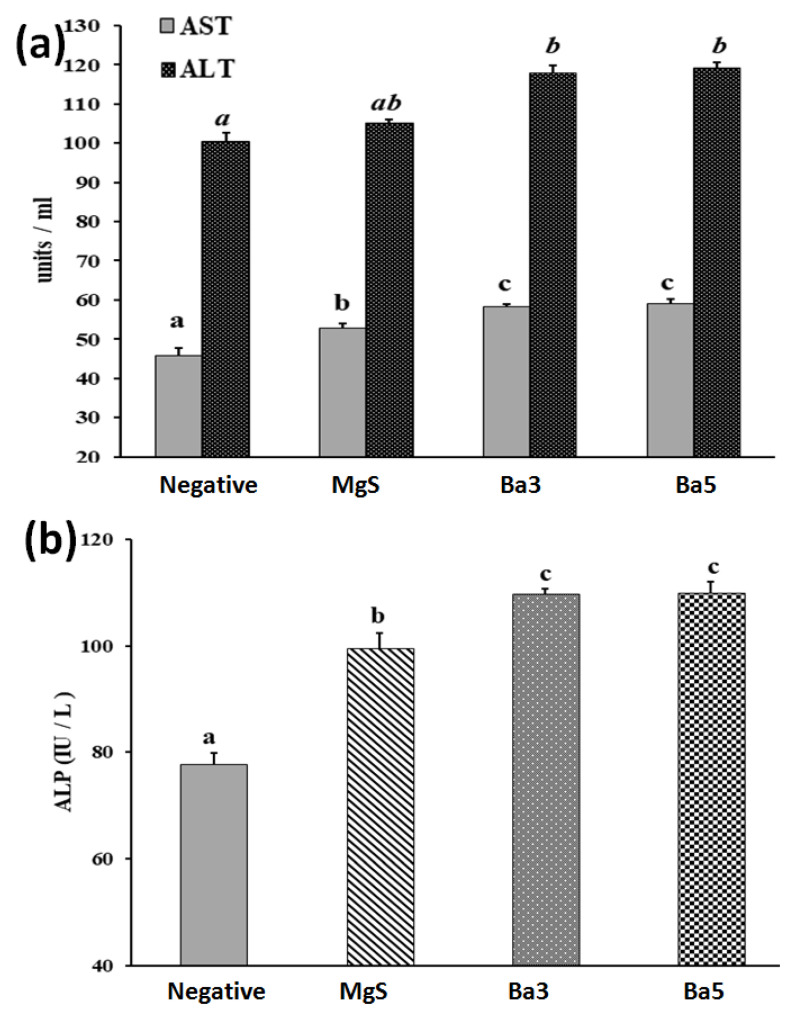
Effect of MgS nanopowders free from and doped with BaO on (**a**) hepatotoxicity indices and (**b**) alkaline phosphatase (ALP) in tibia-fractured rats ofdifferent experimental groups: Sham Control, MgS, MgS doped with 3% BaO (Ba3), MS doped with 5% BaO (Ba5). Data are presented as mean ± SE of mean. Statistical analysis was conducted using ANOVA followed by Duncan test (*n* = 5, *p* ≤ 0.05). Mean with different superscripts (a,b,c) are significant at *p*≤0.05. Bars having the same superscripts are not significantly different from each other, while those having different superscripts are significantly different from each other (ALT: alanine amino transaminase, AST: aspartate amino transaminase and ALP: alkaline phosphatase).

In order to evaluate the hepatotoxic influence and bone-formation potential of three samples (MgS and two BaO-doped nanopowders); serum levels of AST, ALT and ALP were measured as represented in Figure 9a,b. As compared to Sham control rats, ALP was significantly elevated by 28.23% for MgS groups, and 41.38 and 41.59%, respectively, for MgS nanopowders doped with BaO (3 and 5% *w/w*). On the other hand, AST and ALT activities showed a significant increase in MgS (15.58%), and insignificant change regarding ALT activities. Considering BaO-doped groups, AST and ALT activities were significantly elevated by 27.78 and 17.37% for Ba3, and 29.36 and 18.75% for Ba5. These data of transaminases demonstrated that proper performance of the liver was not largely disrupted by the implantation of MgS free from and doped with BaO in tibia-fractured rats, which normally takes place upon bone surgeries. Furthermore, BaO-doped groups (3 and 5% *w/w*) exhibited higher bone-formation potential than that of MS-group, manifested as elevated serum ALP levels.

**Figure 10:**
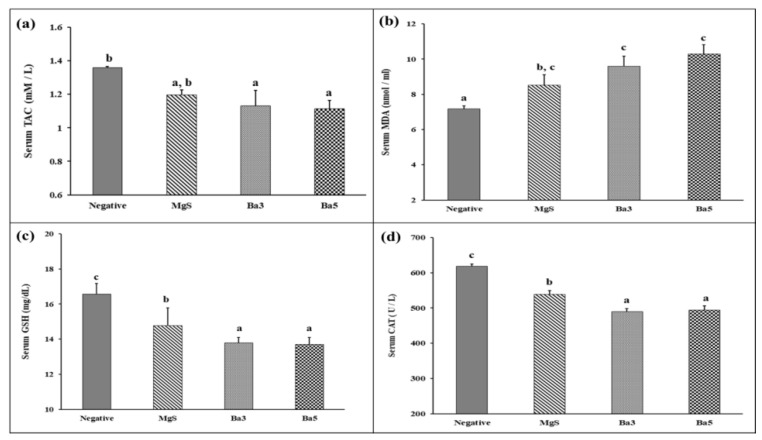
Effect of MgS nanopowders free from and doped with BaO on serum total antioxidant capacity (TAC), serum Malondialdehyde (MDA), glutathione reduced (GSH) and catalase (CAT) in tibia-fractured rats of different experimental groups: Sham Control, MgS, MgS doped with 3% BaO (Ba3) and MS doped with 5% BaO (Ba5). Data are presented as mean ± SE of mean. Statistical analysis was conducted using ANOVA followed by Duncan test (*n* = 5, *p* ≤ 0.05). Means with different superscripts (a, b, c) are significant at *p* ≤ 0.05. Bars having the same superscripts are not significantly different from each other, while those having different superscripts are significantly different from each other. (**a**): TAC, (**b**): MDA, (**c**): GSH and (**d**): CAT.

Moreover, oxidative stress was estimated in different experimental groups through measuring the serum levels of TAC, MDA and GSH and CAT activities, as illustrated in Figure 10a–c. Regarding MgS nanopowders, there was a mild decrease in serum TAC levels (11.76%), along with a significant increase in serum MDA levels (18.84%), while serum GSH and CAT activity showed a mild decrease in MgS group by 10.82 and 12.89%, respectively, as compared to the Sham group. On the other hand, serum TAC levels were significantly decreased by 16.65 and 18%, respectively, for Ba3 and Ba5 groups, as compared to the Sham group. Serum MDA was significantly increased by 33.78 and 43.42%, respectively, for Ba3, and Ba5 groups, as compared to the Sham group. Serum GSH levels and CAT activities were significantly declined by 16.66 and 20.77%, respectively, for Ba3 and 17.33 and 20.12%, respectively, forBa5 groups, as compared to the Sham group. These data show the presence of relative oxidative potential in MgS, Ba3 and Ba5 samples.

**Figure 11:**
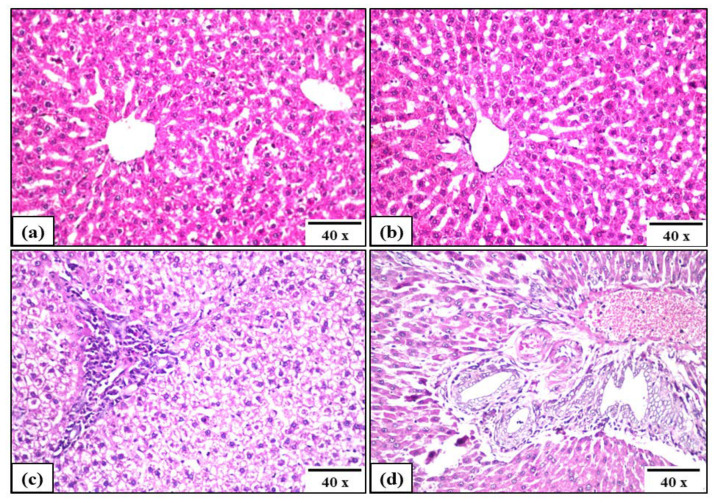
Effect of three MgS free from and doped with BaO on liver histopathology of tibia-fractured rats of different experimental groups: (**a**) sham control, (**b**) MgS,(**c**) MgSdoped with 3% BaO (Ba3) and (**d**) MgS doped with 5% BaO (Ba5).

In addition to the above-mentioned biomarkers, the histopathological analysis of the liver of the treated rats compared to Sham control rats were also evaluated (Figure 11). The Sham control group demonstrated no histopathological alterations and the normal histological structure of the central vein and surrounding hepatocytes in the parenchyma were shown in Figure 11a. Similarly, MgS group showed no histopathological changes, as recorded in Figure 11b. On the other hand, the liver tissues of both Ba3 and Ba5 groups demonstrated a certain degree of hepatic inflammation; the portal area in Ba3 liver tissues demonstrated inflammatory cells infiltration associated with degeneration in the hepatocytes (Figure 11c). While Ba5 liver tissues demonstrated a very minor disruption of the hepatic structure, there were multiple numbers of newly formed bile ductules in the portal area associated with few inflammatory cells (Figure 11d).

#### 3.7.3. Histopathological Analysis of Tibia-Fractured Rats

**Figure 12:**
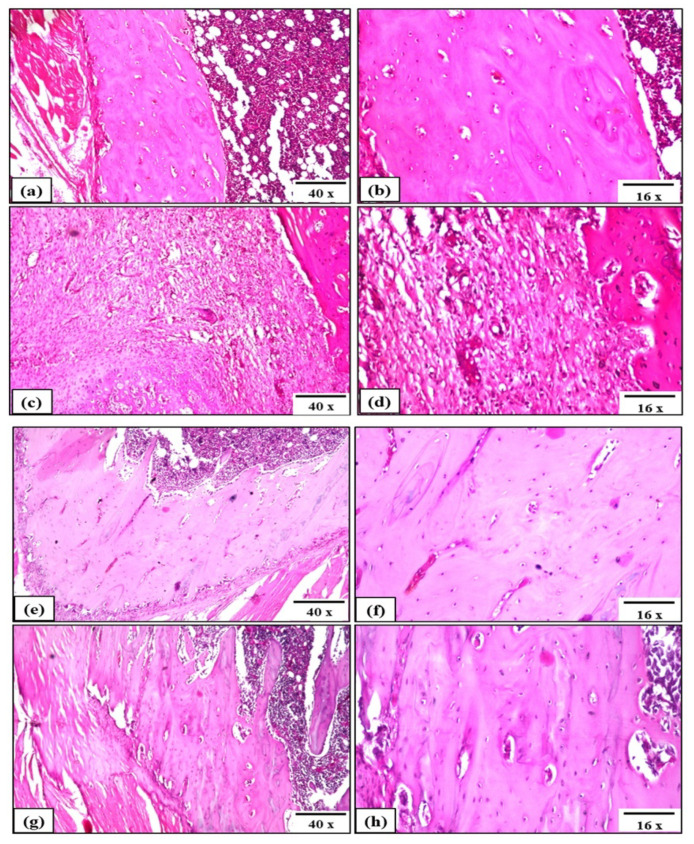
Effect of three MgSs free from and doped with BaO on bone histopathology of tibia-fractured rats of different experimental groups: Sham Control: (**a**,**b**), MgS: (**c**,**d**), MgS doped with 3% BaO (Ba3): (**e**,**f**), MgS doped with 5% BaO (Ba5): (**g**,**h**).

Sham control rats demonstrated no histopathological changes and the normal histological structure of the compact bone with osteoblasts and adjacent bone marrow and covered by periosteum are shown in (Figure 12a,b), while an area of collagen and newly formed blood capillaries replaced the injured bone treated with pure MgS (Figure 12c,d). Likewise, rats treated with Ba3 and Ba5 demonstrated normal bone healing where osteoblasts presence with newly formed capillaries as an indication of bone regeneration of the fractured area (Figure 12e,f) and normal intact histological structure of the osteoblasts adjacent to the bone marrow (Figure 12g,h).

## 4. Discussion

Evaluating the bone-repairing potential of novel MgS nanopowders free from and doped with barium (BaO) in rodent models is a valuable tool in the pre-clinical studies that facilitates estimation of the extent of new bone formation through histological and biomarkers investigations [35]. Herein, this study aimed to compare the bone-healing potential and the hepatotoxic activity of three different nanopowders based on MgS. Magnesium (Mg) is an essential mineral element in bone metabolism that plays a vital role in the bone mineralization through interfering with osteoblast activities [36,37]. Mg ions (Mg^2+^) have been utilized as a dopant in several matrices to ameliorate mechanical function and reduce the rate of matrices degradation [38,39]. Several Mg-containing silicate ceramics are bioactive and attractive for biomedical applications due to their unique biological and mechanical features in addition to their impressive influence on the biomineralization and cell reproduction [40,41,42]. Moreover, divalent cation “Mg^2+^” plays a principal role in bone remodeling and angiogenesis [42,43]. On the other hand, it was evidenced that silicate-based materials increased bone density and bone turnover in osteopenia in ovariectomized rats fed with calcium-reduced diet [44]. In addition, there is a direct association between collagen and “Si”; total bone Si content is directly co-related with age [45], “Si” can enhance the process of bone formation through stimulating up-regulation of collagen type-I [46]; in addition, orthosilicic acid-enriched medium of human osteoblast-like cells enhanced the release of collagen type-I, ALP, and osteocalcin [47]. More interestingly, degradation of Si-containing substitute bone materials did not evoke any signs of cytotoxicity, genotoxicity or carcinogenicity [48,49].

The physicochemical features observed for the MgS nanopowders doped with BaO exhibited significant changes compared to the pure MgS. In particular, the presence and increase in the concentration of BaO had enhanced the crystallization of the achieved nanopowders in relation to their native material as noticed from the XRD results [50]. Moreover, the presence of BaO within the MgS nanopowders was confirmed by the FTIR and the bands related to magnesium presence were diminished with the increment of BaO concentrations, which confirms that the BaO doping was successful on the expense of Mg. Previous researchers proved that the inclusion of different transition metals such as Ba^2+^enhance the physicochemical properties of the parent materials [34,51]. Moreover, the morphology and size of both agglomerated (SEM images in Figure 4) and dispersed nanopowders (TEM images in Figure 6) confirmed the effect of BaO inclusion and concentration on the diameter range of the obtained nanopowders. Fusion of nanopowders that takes place during the formation of the ceramic phases in the current samples was given as the main cause of these observed changes. However, it is worthy to highlight the different size of dopant element (Ba^2+^) and the Mg^2+^(element being partially replaced) might cause great influences on the final sizes of the nanopowders, the ionic radius of Ba^2+^ (r = 1.42 Å), where Mg^2+^ (r = 0.57 Å) [52]. Accordingly, the surface areas of these nanopowders were relatively affected by the existence and concentration of BaO, which could also be dueto the difference between the ions’ radii, as mentioned on the above.

BaO doping exhibited a positive effect of the mechanical properties of MgS materials, which is consistent with the early reported works [22,33]. Furthermore, notable antibacterial capacity against the under investigated bacterial strains were observed, even for the BaO-free sample. This reveals the important role of Ba^2+^ and Mg^2+^ leached ions in the tested media. The role of Mg^2+^in the prevention of bacterial growth was explored in several articles [52,53]. Normally, the antibacterial mechanism process initiated by magnesium or magnesium-containing nanoparticles in previous research [54,55] suggested that the bacterial cell’s membrane is damaged, bringing about a spillage of intracellular substance and in the long run the death of bacterial cells. Moreover, its alkalization role, in which the leakage of Mg^2+^ ions occurs due to magnesium erosion, was raised several times as a significant component of antibacterial capability. In order to separate the effect of pH from the MgO concentration, a recent study used different concentrations of Mg^2+^ and fixed the pH value (8.0 ± 0.1) during the experiment and found that the higher concentration of Mg^2+^ possess a strong effect against the utilized bacterial strains and the damage to the bacterial cell membrane was due to the great osmotic stress caused by the higher concentration of Mg^2+^ [56]. Many transition metals in nanoscale demonstrated bactericidal activity against different bacterial species; thus, their mechanisms of action were explained due to several reasons, such as disruption of cell membrane and/or arrest of the cell cycle due to generation of reactive oxygen species (ROS) [57]. This explanation is of a great potential to be the suggested mechanism for Ba^2+^ bactericidal activity.

The MgS nanopowders exerted an effective bone healing potential without stimulating nanotoxic potential (Figure 12b); MgS nanopowders are biocompatible and non-hepatotoxic. Therefore, MgS nanopowders could be a promising candidate as bone cement for rapid healing of fractures in large bones. The implanted materials in bone should be biocompatible and non-toxic to different body organs, including the liver. Through the evaluation of liver function biomarkers (Figure 9), AST and ALT activities were relatively increased in Ba3 and Ba5 groups, while demonstrated a mild increase in MgS group, as compared to Sham control. These elevated hepatic activities in BaO-doped groups might be ascribed to the uptake of Ba-NPs by hepatocytes and Kupffer cells, due to the decreased size of Ba^2+^ released nanoparticles for both samples, i.e., Ba3 and Ba5 (45–50 nm and 20–27 nm, respectively), while MgS nanopowders exhibited the higher range of leaked particles (50–60 nm). However, elevated AST and ALT activities were not associated with hepatotoxicity, as demonstrated by the histopathological investigation of the liver (Figure 11b–d). This elevated serum levels of transaminases might be demonstrated as a normal biological response against exposure to nanoparticles, as explained previously [58].

Furthermore, ALP activity has been identified in different tissues, including the liver, bone, kidney and white blood cells. However, significant serum secretion has been linked to bone and liver tissues [59]. Therefore, the ALP secretion into the blood indicates either liver disorder or bone disease. As compared to Sham group, ALP demonstrated the least activity in MgS group indicates the least mineralization potential of MgS formulation (28.23%) and a higher activity (about 42%) for both Ba3 and Ba5 groups, signifying the higher calcium deposition and mineralization potential exerted by MgS BaO doped groups. This elevated ALP activity could be explained by the ability of active osteoblasts “bone-forming cells”, during the maturation phase of healing process, to secrete high amounts of ALP and osteocalcin, which are essential for bone mineralization. The bone healing process is characterized by increased ALP activity, while bone fracture induces a decrease in ALP activity [15]. Accordingly, specific serum biomarkers of bone formation such as ALP could be useful for evaluating of the progress of bone healing process. Similarly, doping MgS nanopowders with different concentrations of “BaO” enhanced a mild oxidative stress, through increasing serum MDA levels and reducing serum of TAC and GSH levels, along with a declined serum CAT activity, which could be explained as a normal physiological defense mechanism against Ba^2+^ NPs that does not elicit nanotoxicity.

Consistent with the results of SEM and TEM images, surface area parameters were evaluated for all the prepared samples using BET surface area (m²/g) measurements, as listed in Table 2. Major negative effects for BaO presence and concentration were observed, where the surface area was decreased from 122.63 ± 0.54 m²/g for pure MgS to 75.20 ± 0.41 and 64.66 ± 0.37, respectively, for MgS doped with 3 and 5% *w/w* BaO, demonstrating a smaller particle size, which is supposed to possess a higher surface area value. This could be explained by the presence of BaO, which increased the cementing property of these materials, which had increased their agglomeration and decreased their surface area. We found that the agglomerate sizes were influenced by particle concentration: the higher the concentration, the larger the hydrodynamic diameter. The pore diameter was increased form 10.78 ± 0.54 for MgS to 11.08 ± 0.62 for Ba3 and 11.10 ± 0.47 for Ba5. Herein, we could find that doping with BaO elicited a relative hepatic inflammation and oxidative stress. Our study runs in agreement with a previous study that reported that the addition of “BaO” in porous ceramic foam did not impart any toxic property to the osteoblast cells [60].

Moreover, the existence of the “Ba^2+^” concentration increases the crystallinity degree and the particles fusions, as confirmed by the XRD results, which caused the increment of the particle size of Ba3- and Ba5-doped nanopowders. It can be noted that the particle size and crystallinity degree of the prepared nanopowders are highly affected by the presence of BaO dopant and its concentration. It was confirmed that non-spherical nanomaterials may exhibit different toxicity compared with spherically shaped nanomaterials of the same composition due to the varying local charge density [61]. In addition, we could deduce that the variation in the potential of Ba^2+^NPs to provoke inflammation and oxidative stress is size-dependent; small-sized NPs exhibit a higher degree of degradation, and consequently, a higher tendency of agglomeration and bioreactivity as well.

Comparing the histopathological features of both the Sham control and the treated tibia-fractured groups after five weeks, it could be observed that tibia treated with the three MgSs free from and doped with BaO demonstrated an area of collagen and cartilaginous tissues restoration, indicating complete bone reconstruction with normal architecture of bone tissue and remodeling in a very short time, as compared to the natural healing process. Therefore, the three nanopowders exerted an effective bone healing with signs of osteoblast proliferation and absence of the fracture gap. For a deeper differentiation between the examined formulations of the nanopowders in the healing process, histopathological investigation of liver tissues was performed to assess the degree of hepatic inflammation. Sham and MgS groups demonstrated normal histological structure of hepatocytes, while BaO-doped samples (Ba3 and Ba5 groups) demonstrated slightly altered structure of hepatocytes and hepatic inflammation indicating mild increase in ALT and AST, which are supported with histopathological examination of liver tissues; this could be explained by the potential of these nanopowders to provoke different degrees of in vivo inflammation and oxidative effects. The biochemical, histopathological and mechanical results suggest that three nanopowders could effectively ameliorate the bone healing process through stimulating osteoblast cells; the bone healing potential of BaO-doped MgS nanopowders was more potent as evidenced by elevated ALP activities. However, this bone healing activity was associated with induction of acceptable oxidative response and hepatic inflammation. Therefore, we could deduce that BaO-doped MgS nanopowders demonstrated a safe toxicological profile and could be used as bone fracture healing materials.

The important features of crystalline implants such as the degrees of biocompatibility and biodegradation/resorption were seen to be affected by several causes including the degree of crystallinity and the phase compositions as it was stressed out earlier [62]. Moreover, the recorded mechanical properties of the BaO-doped MgS are relatively close to the mechanical properties of trabecular bone that were revealed earlier [63]. These results reflect the impressive influence of BaO doping in MgS nanopowders, which are highly correlated to their crystallinity degree, consistent with previous works [22,33].

Ba does not cause a bioaccumulation in human tissues; average Ba levels in body tissues are determined to be about 20 mg [64]. Ba poisoning is rather uncommon [65]; it seems to be a non-carcinogenic metal, unlike other heavy metals. Moreover, the U.S. Environmental Protection Agency (US EPA) concluded that Ba is not likely to be carcinogenic to humans [66]. Biomonitoring reports have been conducted on urinary concentration of Bain Ba-exposed mice to investigate the potential influence on human health [67]. Ba exposure can be classified into chronic (more than one year), intermediate (15–364 days) and acute (<14 days). Intermediate exposure was investigated for inhalation and oral routes in animals, while chronic exposures were examined orally [68]. Accordingly, Ba exposure in this study belongs to intermediate type (5 weeks); therefore, the biomarkers of liver function and oxidative stress were estimated to evaluate the toxic potential of Ba, and our findings revealed the moderate elevation of transaminases and oxidative response was acceptable, as a normal response, and that BaO-doped cements showed good biocompatibility. Furthermore, a previous study proposed that adding Ba permits the radiopacity and strength of bone cement to be employed in clinical application [68]. Therefore, further research is required to determine the potential health consequences of using BaO-doped bone cements before its clinical application.

BaO-doped MgS nanopowders are well tolerated and induce neither hepatotoxicity nor oxidative injury, suggesting their potential for bone healing applications. Therefore, future studies should be oriented to develop nanomaterials that are capable of mimicking the structural and mechanical features of bone and enhancing vascularization or new bone formation, as well as establishing in vivo models that could be employed to compare between different formulations of bone fractures healing materials, and to finally produce biomaterials with superior properties for bone healing through enhancing bone regeneration and providing mechanical strength and support for the fractured bones, especially for those cases demanding prolonged treatment.

## 5. Conclusions

In the present work, for the firsttime, in vivo biocompatibility of pure MgS and BaO-doped MgS nanopowders were reported alongside with their ability to promote new bone formation. The crystallinity and the size of BaO-doped MgS nanopowders were increased compared to the pure MgS, as confirmed by XRD, SEM and TEM, while the surface area diminished after BaO doping. However, these physical and morphological changes did not affect the final chemical features of the nanopowders as confirmed by FTIR results. In addition, increased mechanical and antibacterial activities were perceived for higher doping concentrations of BaO. The BaO-doped MgS samples showed a higher tendency to form full new bone that affirms the biocompatibility of theses nanomaterials. Although sample Ba7 demonstrated a higher regeneration rate, the biosafety tests in this work showed normal biomarkers for lower concentrations of BaO (Ba3 and Ba5). Furthermore, the nanopowders were biocompatible as confirmed from the biochemical and histopathological assays. Therefore, MgS and BaO-doped MgS nanopowders show a capability to be used as a regenerative biomaterial for bone fracture healing application. In the future, the clearance of the current nanopowders will be investigated over long periods after implantation (prior to their clinical trials).

## Figures and Tables

**Table 1:** Samples compositions (%) adopted in this study.

Sample	SiO_2_	MgO	BaO
**MgS**	50	50	--
**Ba3**	50	47	3
**Ba5**	50	45	5
**Ba7**	50	43	7

**Table 2:** Surface area parameters for the MgS cement before and after BaO doping.

Sample	BET Surface Area (m²/g)	Pore Volume (cm³/g)	Pore Diameter (nm)
MgS	122.63 ± 0.54	0.41± 0.04	10.78 ± 0.54
Ba3	75.20 ± 0.41	0.22 ± 0.03	11.08 ± 0.62
Ba5	64.66 ± 0.37	0.19 ± 0.05	11.10 ± 0.47
Ba7	37.95 ± 0.30	0.13 ± 0.04	12.81 ± 0.83

## Data Availability

Data will be madeavailable upon request.

## References

[1] Sabet F.A., Najafi A.R., Hamed E., Jasiuk I. (2016). Modelling of bone fracture and strength at different length scales: A review. Interface Focus.

[2] Javaid M.K., Kyer C., Mitchell P.J., Chana J., Moss C., Edwards M.H., McLellan A.R., Stenmark J., Pierroz D.D., Schneider M.C. (2015). Exco, effective secondary fracture prevention: Implementation of a global benchmarking of clinical quality using the IOF Capture the Fracture^®^ Best Practice Framework tool. Osteoporos. Int..

[3] Marmor M., Alt V., Latta L., Lane J., Rebolledo B., Egol K.A., Miclau T. (2015). Osteoporotic fracture care: Are we closer to gold standards?. J. Orthop. Trauma.

[4] Marsell R., Einhorn T.A. (2011). The biology of fracture healing. Inj. Int. J. Care Inj..

[5] Egermann M., Lill C.A., Griesbeck K., Evans C.H., Robbins P.D., Schneider E., Baltzer A.W. (2006). Effect of BMP-2 gene transfer on bone healing in sheep. Gene Ther..

[6] Phillips A.M. (2005). Overview of the fracture healing cascade. Injury.

[7] Tautzenberger A., Kovtun A., Ignatius A. (2012). Nanoparticles and their potential for application in bone. Int. J. Nanomed..

[8] Dimitriou R., Jones E., McGonagle D., Giannoudis P.V. (2011). Bone regeneration: Current concepts and future directions. BMC Med..

[9] Aydin A., Memisoglu K., Cengiz A., Atmaca H., Muezzinoglu B., Muezzinoglu U.S. (2012). Effects of botulinum toxin a on fracture healing in rats: An experimental study. J. Orthop. Sci..

[10] LaStayo P.C., Winters K.M., Hardy M. (2003). Fracture healing: Bone healing, fracture management and current concepts related to the hand. J. Hand Ther..

[11] Wang Q., Yan J., Yang J., Li B. (2016). Nanomaterials promise better bone repair. Mater. Today.

[12] Perez R.A., Won J.-E., Knowles J.C., Kim H.-W. (2013). Naturally and synthetic smart composite biomaterials for tissue regeneration. Adv. Drug Deliv. Rev..

[13] Balansundarum G., Storey D.M., Webster T.J. (2014). Novel nano-rough polymers for cartilage tissue engineering. Int. J. Nanomed..

[14] Mabrouk M., Das D.B., Salem Z.A., Beherei H.H. (2021). Nanomaterials for Biomedical Applications: Production, Characterisations, Recent Trends and Difficulties. Molecules.

[15] Florence N.T., Huguette S.T.S., Hubert D.J., Raceline G.K., Desire D.D.P., Pierre K., Theophile D. (2017). Aqueous extract of *Peperomia pellucida* (L.) HBK accelerates fracture healing in Wistar rats. BMC Complement. Altern. Med..

[16] Chen Z., Kang L., Meng Q.Y., Liu H., Wang Z., Guo Z., Cui F.Z. (2014). Degradability of injectable calcium sulfate/mineralized collagen-based bone repair material and its effect on bone tissue regeneration. Mater. Sci. Eng. C Mater. Biol. Appl..

[17] Guo Z., Liu X.-M., Ma L., Li J., Zhang H., Gao Y.-P., Yuan Y. (2013). Effects of particle morphology, pore size and surface coating of mesoporous silica on Naproxen dissolution rate enhancement. Colloids Surf. B Biointerfaces.

[18] Lin K., Liu Y., Huang H., Chen L., Wang Z., Chang J. (2015). Degradation and silicon excretion of the calcium silicate bioactive ceramics during bone regeneration using rabbit femur defect model. J. Mater. Sci. Mater. Med..

[19] Khan A.F., Saleem M., Afzal A., Ali A., Khan A., Khan A.R. (2014). Bioactive behavior of silicon substituted calcium phosphate based bioceramics for bone regeneration. Mater. Sci. Eng. C Mater. Biol. Appl..

[20] Zhou H., Wu X., Wei J., Lu X., Zhang S., Shi J., Liu C. (2011). Stimulated osteoblastic proliferation by mesoporous silica xerogel with high specific surface area. J. Mater. Sci. Mater. Med..

[21] Bose S., Fielding G., Tarafder S., Bandyopadhyay A. (2013). Understanding of dopant-induced osteogenesis and angiogenesis in calcium phosphate ceramics. Trends Biotechnol..

[22] Alshemary A.Z., Goh Y.-F., Akram M., Kadir M.R.A., Hussain R. (2015). Barium and fluorine doped synthetic hydroxyapatite: Characterization and in-vitro bioactivity analysis. Sci. Adv. Mater..

[23] Zamora L.L., Perez-Gracia M.T. (2012). Using digital photography to implement the McFarland method. J. R. Soc. Interface.

[24] Anitha S., Muthukumaran S. (2020). Structural, optical and antibacterial investigation of La, Cu dual doped ZnO nanoparticles prepared by co-precipitation method. Mater. Sci. Eng. C Mater. Biol. Appl..

[25] Bigham-Sadeg A., Karimi I., Hoseini F., Oryan A., Sharifi S., Pakzad A. (2018). Effects of honey and hydroxyapatite on bone healing in rats. Trauma Mon..

[26] Reitman S., Frankel S. (1957). A colorimetric method for the determination of serum glutamic oxalacetic and glutamic pyruvic transaminases. Am. J. Clin. Pathol..

[27] Belfield A., Goldberg D.M. (1971). Normal ranges and diagnostic value of serum 5′nucleotidase and alkaline phosphatase activities in infancy. Arch. Dis. Child..

[28] Koracevic D., Koracevic G., Djordjevic V., Andrejevic S., Cosic V. (2001). Method for the measurement of antioxidant activity in human fluids. J. Clin. Pathol..

[29] Ohkawa H., Ohishi N., Yagi K. (1979). Assay for lipid peroxides in animal tissues by thiobarbituric acid reaction. Anal. Biochem..

[30] Tekin S., Seven E. (2021). Assessment of serum catalase, reduced glutathione, and superoxide dismutase activities and malondialdehyde levels in keratoconus patients. Eye.

[31] Krishna H., Avinash K., Shivakumar A., Al-Tayar N.G.S., Shrestha A.K. (2021). A quantitative method for the detection and validation of catalase activity at physiological concentration in human serum, plasma and erythrocytes. Spectrochim. Acta A Mol. Biomol. Spectrosc..

[32] Bancroft J.D., Stevens A., Turner D.R. (1996). Theory and Practice of Histological Techniques.

[33] Myat M.-H., Noor A.-F.M., Kawashita M., Ismail Y.M.B. (2020). Enhanced sinterability and in vitro bioactivity of barium-doped akermanite Ceramic. Ceram. Int..

[34] Sun Z., Xinhui D., Srinivasakannan C., Liang J. (2018). Preparation of magnesium silicate/carbon composite for adsorption of rhodamine B. RSC Adv..

[35] Peric M., Dumic-Cule I., Grcevic D., Matijasic M., Verbanac D., Paul R., Grgurevic L., Trkulja V., Bagi C.M., Vukicevic S. (2015). The rational use of animal models in the evaluation of novel bone regenerative therapies. Bone.

[36] Kanasan N., Adzila S., Koh C.T., Rahman H.A., Panerselvan G. (2019). Effects of magnesium doping on the properties of hydroxyapatite/sodium alginate biocomposite. Adv. Appl. Ceram..

[37] Schatkoski V.M., doMontanheiro A., de Menezes T.L., Pereira B.R.C., Rodrigues R.M., Rodrigues K.F., Ribas R.G., da Silva D.M., Thim G.P. (2021). Current advances concerning the most cited metal ions doped bioceramics and silicate-based bioactive glasses for bone tissue engineering. Ceram. Int..

[38] Munirathinam B., Jaladurgam N.R., Magesh J., Narayanan R., Mol J.M., Neelakantan L. (2019). Improved corrosion protection of titanium implant material by crystallographic texturing of Sr doped calcium phosphate electrodeposits. Thin Solid Films.

[39] Tabia Z., El Mabrouk K.M., Nouneh B.K. (2019). Mesoporous bioactive glass nanoparticles doped with magnesium: Drug delivery and acellular in vitro bioactivity. RSC Adv..

[40] Ni S., Chou L., Chang J. (2007). Preparation and characterization of forsterite (Mg_2_SiO_4_) bioceramics. Ceram. Int..

[41] Oudadesse H., Martin S., Derrien A., Lucas-Girot A., Cathelineau G., Blondiaux G. (2004). Determination of Ca, P, Sr and Mg in the synthetic biomaterial aragonite by NAA. J. Radioanal. Nucl. Chem..

[42] Diba M., Goudouri O.M., Tapia F., Boccaccini A.R. (2014). Magnesium-containing bioactive polycrystalline silicate-based ceramics and glass-ceramics for biomedical applications. Curr. Opin. Solid State Mater. Sci..

[43] Zhai W., Lu H., Chen L., Lin X., Huang Y., Dai K., Naoki K., Chen G., Chang J. (2012). Silicate bioceramicsinduce angiogenesis during bone regeneration. Acta Biomater..

[44] Nielsen F.H. (2014). Update on the possible nutritional importance of silicon. J. Trace Elem. Med. Biol..

[45] Jugdaohsingh R., Pedro L.D., Watson A., Powell J.J. (2014). Silicon and boron differ in their localization and loading of bone. Bone Rep..

[46] Shie M.-Y., Ding S.-J., Chang H.-C. (2011). The role of silicon in osteoblast-like cell proliferation and apoptosis. Acta Biomater..

[47] Reffitt D.M., Ogston N., Jugdaohsingh R., Cehung H.F.J., Evans R.P.H., Thompson R.P.H., Powell J.J., Hampson G.N. (2003). Orthosilic acid stimulates collagen type I synthesis and osteoblastic differentiation in human osteoblast-like cells in vitro. Bone.

[48] European Food Society Authority (EFSA) (2004). Opinion of the Scientific Panel on Dietetic Products, Nutrition and Allergies on a request from the Commission related to the tolerable upper intake level of silicon. EFSA J..

[49] Götz W., Tobiasch E., Witzleben S., Schulze M. (2019). Effects of silicon compounds on biomineralization, osteogenesis, and hard tissue formation. Pharmaceutics.

[50] Yonesaki Y., Takei T., Kumada N., Kinomura N. (2009). Crystal structure of Eu^2+^-doped M_3_MgSi_2_O_8_ (M: Ba, Sr, Ca) compounds and their emission properties. J. Solid State Chem..

[51] Joseph T., Sebastian M.T. (2010). Microwave Dielectric Properties of (Sr_1−*x*_A*_x_*)_2_(Zn_1−x_B_x_)Si_2_O_7_ Ceramics (A = Ca, Ba and B = Co, Mg, Mn, Ni). J. Am. Ceram. Soc..

[52] Singh R.K., Kannan S. (2014). Synthesis, Structural analysis, Mechanical, antibacterial and Hemolytic activity of Mg^2+^ and Cu^2+^ co-substitutions in β-Ca_3_(PO_4_)_2_. Mater. Sci. Eng. C.

[53] Guo Z., Zhang Z., Zhang N., Gao W., Li J., Pu Y., He B., Xie J. (2022). A Mg^2+^/polydopamine composite hydrogel for the acceleration of infected wound healing. Bioact. Mater..

[54] Luque-Agudo V., Fernández-Calderón M.C., Pacha-Olivenza M.A., Pérez-Giraldo C., Gallardo-Moreno A.M., González-Martín M.L. (2020). The role of magnesium in biomaterials related infections. Colloids Surf. B Biointerfaces.

[55] Hickey D.J., Muthusamy D., Webster T.J. (2017). Electrophoretic deposition of MgO nanoparticles imparts antibacterial properties to poly-L-lactic acid for orthopedic applications. J. Biomed. Mater. Res. Part A.

[56] Rodríguez-Sánchez J., Pacha-Olivenza M., González-Martín M. (2019). Bactericidal effect of magnesium ions over planktonic and sessile *Staphylococcus epidermidis* and *Escherichia coli*. Mater. Chem. Phys..

[57] Tong G., Du F., Wu W., Wu R., Liu F., Liang Y. (2013). Enhanced reactive oxygen species (ROS) yields and antibacterial activity of spongy ZnO/ZnFe_2_O_4_ hybrid micro-hexahedra selectively synthesized through a versatile glucose-engineered co-precipitation/annealing process. J. Mater. Chem. B.

[58] Mabrouk M., Fouad G.I., El-Sayed S.A.M., Rizk M.Z., Beherei H.H. (2021). Hepatotoxic and Neurotoxic Potential of Iron Oxide Nanoparticles in Wistar Rats: A Biochemical and Ultrastructural Study. Biol. Trace Elem. Res..

[59] Mahdy E.A., Sahbal K.M., Mabrouk M., Beherei H.H., Abdel-Monem Y.K. (2021). Enhancement of glass-ceramic performance by TiO_2_ doping: In Vitro cell viability, proliferation, and differentiation. Ceram. Int..

[60] Ball J.P., Mound B.A., Nino J.C., Allen J.B. (2014). Biocompatible evaluation of barium titanate foamed ceramic structures for orthopedic applications. J. Biomed. Mater. Res. Part A.

[61] Wani M.Y., Hashim M.A., Nabi F., Malik M.A. (2011). Nanotoxicity: Dimensional and morphological concerns. Adv. Phys. Chem..

[62] Kamitakahara M., Ohtsuki C., Miyazaki T. (2008). Review paper: Behavior of ceramic biomaterials derived from tricalcium phosphate in physiological condition. J. Biomater. Appl..

[63] Misch C.E., Qu Z., Bidez M.W. (1999). Mechanical properties of trabecular bone in the human mandible: Implications for dental implant treatment planning and surgical placement. J. Oral Maxillofac. Surg..

[64] Kravchenko J., Darrah T.H., Miller R.K., Lyerly H.K., Vengosh A. (2014). A review of the health impacts of barium from natural and anthropogenic exposure. Environ. Geochem. Health.

[65] Emsley J. (1998). The Elements.

[66] Poddalgoda D., Macey K., Assad H., Krishnan K. (2017). Development of biomonitoring equivalents for barium in urine and plasma for interpreting human biomonitoring data. Regul. Toxicol. Pharmacol..

[67] Peana M., Medici S., Dadar M., Zoroddu M.A., Pelucelli A., Chasapis C.T., Bjørklund G. (2021). Environmental barium: Potential exposure and health-hazards. Arch. Toxicol..

[68] Makita M., Yamakado K., Nakatsuka A., Takaki H., Inaba T., Oshima F., Katayama H., Takeda K. (2008). Effects of barium concentration on the radiopacity and biomechanics of bone cement: Experimental study. Radiat. Med..

